# The influence of mouth opening on pharyngeal pressure loss and its underlying mechanism: A computational fluid dynamic analysis

**DOI:** 10.3389/fbioe.2022.1081465

**Published:** 2023-01-09

**Authors:** Bin Hu, Guoping Yin, Song Fu, Baoshou Zhang, Yan Shang, Yuhuan Zhang, Jingying Ye

**Affiliations:** ^1^ Department of Otolaryngology-Head Neck Surgery, Beijing Tsinghua Changgung Hospital, School of Clinical Medicine, Tsinghua University, Beijing, China; ^2^ Sleep Medicine Center, Beijing Tsinghua Changgung Hospital, School of Clinical Medicine, Tsinghua University, Beijing, China; ^3^ School of Aeronautics and Astronautics, Tsinghua University, Beijing, China

**Keywords:** pressure loss, mouth breathing, obstructive sleep apnea, computer tomography, computer simulation, pharynx

## Abstract

**Objective:** During inspiration, mechanical energy generated from respiratory muscle produces a negative pressure gradient to fulfill enough pulmonary ventilation. The pressure loss, a surrogate for energy loss, is considered as the portion of negative pressure without converting into the kinetic energy of airflow. Mouth opening (MO) during sleep is a common symptom in patients with obstructive sleep apnoea-hypopnea syndrome (OSAHS). This study aimed to evaluate the effects of mouth opening on pharyngeal pressure loss using computational fluid dynamics (CFD) simulation.

**Methods:** A total of four subjects who were morphologically distinct in the pharyngeal characteristics based on Friedman tongue position (FTP) grades were selected. Upper airway computed tomography (CT) scan was performed under two conditions: Mouth closing (MC) and mouth opening, in order to reconstruct the upper airway models. computational fluid dynamics was used to simulate the flow on the two different occasions: Mouth closing and mouth opening.

**Results:** The pharyngeal jet was the typical aerodynamic feature and its formation and development were different from mouth closing to mouth opening in subjects with different Friedman tongue position grades. For FTP I with mouth closing, a pharyngeal jet gradually formed with proximity to the velopharyngeal minimum area plane (plane_Amin_). Downstream the plane_Amin_, the jet impingement on the pharyngeal wall resulted in the frictional loss associated with wall shear stress (WSS). A rapid luminal expansion led to flow separation and large recirculation region, corresponding to the interior flow loss. They all contributed to the pharyngeal total pressure loss. While for FTP I with mouth opening, the improved velopharyngeal constriction led to smoother flow and a lower total pressure loss. For FTP IV, the narrower the plane_Amin_ after mouth opening, the stronger the jet formation and its impingement on the pharyngeal wall, predicting a higher frictional loss resulted from higher WSS. Besides, a longer length of the mouth opening-associated constant constrictive segment was another important morphological factor promoting frictional loss.

**Conclusion:** For certain OSAHS patients with higher Friedman tongue position grade, mouth opening-related stronger jet formation, more jet breakdown and stronger jet flow separation might contribute to the increased pharyngeal pressure loss. It might require compensation from more inspiratory negative static pressure that would potentially increase the severity of OSAHS.

## Introduction

Mouth opening (MO) during sleep is one of the common symptoms in patients with obstructive sleep apnoea-hypopnea syndrome (OSAHS). Humans preferentially breathe *via* the nose route that serves important physiological functions, including humidification, heating, and filtration. However, OSAHS patients with high nasal resistance tend to spend a significant fraction of sleep-time breathing with MO as an alternative route to allow adequate airflow (Fitzpatrick et al., 2003). The upper airway between hard palate and hyoid resembles a “non-rigid pipe” with a soft anterior and lateral wall, which is surrounded by soft palate, tongue, tonsils, parapharyngeal fat pad, etc. The revolving mandible, resulting from MO, compresses the anterior and lateral pharyngeal soft tissue and is responsible for the narrower pharyngeal lumen (Ayuse et al., 2004; Hu et al., 2018). It triggers a vicious cycle that the increased pharyngeal collapsibility due to the compensatory oral breathing route could contribute to the further increase of apnoea and hypopnea (Koutsourelakis et al., 2006). MO has been previously identified as an independent factor contributing to the severity of OSAHS (Fitzpatrick et al., 2003; Ayuse et al., 2004; Koutsourelakis et al., 2006; Friedman et al., 2011; Hu et al., 2018).

Three-dimensional (3D) computed tomography (CT) and magnetic resonance imaging (MRI) are commonly used to evaluate the morphological characteristics of the upper airway and to predict the pharyngeal collapsibility and the severity of OSAHS (Iwatani et al., 2013; Hu et al., 2018). Imaging studies provided precious information about pharyngeal geometry, such as diameter, cross-sectional area, and volume (Iwatani et al., 2013; Hu et al., 2018). As confirmed in our previous imaging study, MO not only caused a stenosis of oropharyngeal lumen in almost all subjects and a more narrowing velopharyngeal lumen especially for subjects with a higher Friedman tongue position (FTP) grade, but also induced the inferior movement of hyoid bone along with an elongated airway segment (Hu et al., 2018). It is widely accepted that the geometric morphology of upper airway determines its aerodynamic characteristics, and the abnormal airflow dynamics may lead to impaired airway ventilation. Exploration of the MO-related pharyngeal morphological variations and the relevant internal airflow changes is crucial for understanding the pathophysiology underlying the relationship between MO and OSAHS.

Recently, computational fluid dynamics (CFD) simulation has been increasingly utilized based on reconstruction of 3D CT images of upper airway to acquire aerodynamic parameters, such as pressure distribution, airflow velocity profile, turbulence intensity, wall shear stress (WSS), and airway resistance (Jeong et al., 2007; Lin et al., 2007; Mihaescu et al., 2011; Sul et al., 2014; Chen et al., 2018; Na et al., 2019; Taherian et al., 2019; Yeom et al., 2019; Feng et al., 2021). Breathing is a complex process, which can be divided into three phases: inspiration, post-inspiration, and active expiration (Malheiros-Lima et al., 2020). During inspiration, mechanical energy generated from the diaphragm contraction produces an increased magnitude of negative pressure between intrathoracic pressure and atmospheric pressure to fulfill enough pulmonary ventilation for gas exchange (Supinski et al., 2018; Harlaar et al., 2021). The negative pressure is considered as a mediator for mechanical energy, originating from respiratory muscle and converting into the kinetic energy of airflow from anterior nostril to pulmonary alveoli during inspiration (Dominelli et al., 2018; Supinski et al., 2018; Kelley et al., 2019). The morphology and inner space of airway continuously change from anterior nostril to pulmonary alveoli. In a certain segment of airway, the luminal pressure should be proportionally distributed, and the airway flow patterns and resistance may therefore be determined by the regional morphological characteristics (Qi et al., 2014; Sul et al., 2014; Na et al., 2019). We proposed the pharyngeal aerodynamics, including pharyngeal pressure distribution, that would change due to the MO-associated reshaped pharyngeal lumen. Besides, MO induced a different tendency in morphological changes of velopharyngeal and/or oropharyngeal segments for subjects with different phenotypes of the relative position of tongue to soft palate that could be defined by the FTP grading system according to our previous study (Hu et al., 2018). Then, the variation tendency of the pharyngeal negative pressure distribution from baseline to MO might vary in subjects with different FTP grades. Nevertheless, no study has yet concentrated on the velopharyngeal and oropharyngeal aerodynamic changes associated with MO using numerical simulation to support this hypothesis.

In the present study, numerical simulation was performed using the 3D anatomically accurate subject-specific upper airway models under the inspiratory conditions with a certain fixed flow rate. The variations of aerodynamic characteristics, including pressure distribution, velocity, airway resistance, turbulence kinetic energy (TKE), and WSS in the velopharyngeal and oropharyngeal segments from baseline to MO were analyzed in subjects with different FTP grades. This study was designed to evaluate the influence of MO on the pharyngeal pressure loss and explore its underlying mechanism. The results would reveal the mediating role of the variations of pharyngeal aerodynamics associated with MO and enhance our understanding about the relationship between the changes of MO-related pharyngeal anatomical features and the severity of OSAHS in patients with habitual MO during sleep.

## Methods

### Subjects

From January 2015 to December 2021, more than 300 subjects who were admitted to the ENT Department or Sleep Medicine Center of Beijing Tsinghua Changgung Hospital were recruited to set up a database, and the following data were collected: demographic characteristics, FTP grades, overnight polysomnography (PSG), CT scan of the upper airway in the baseline mouth closing (MC) position and in the MO position, etc. The inclusion criteria were as follows: 1) apnea-hypopnea index (AHI) > 5 times/h; 2) frequent MO during sleep that was ascertained by patient’s bed-partner. The exclusion criteria were as follows: 1) patients who were aged younger than 18 years old; 2) patients with a history of upper airway surgeries. All enrolled subjects were informed that two successive CT scans might increase the potential risk associated with radiation.

The following subjects were excluded in the process of constructing the upper airway models regardless of being in MC or in MO: 1) the pharyngeal airway was extremely narrow for model reconstruction and grid generation; 2) the continuity of reconstructed pharyngeal airway model was interrupted due to the lower local CT imaging resolution; 3) the bilateral or unilateral nasal common meatus disappeared owing to nasal turbinate hypertrophy and/or deviated septum, and then, nasal flow could not be simulated using CFD.

In the present study, four subjects who were morphologically quite distinct in the pharyngeal characteristics based on FTP grading were selected from the database. The demographic and polysomnographic data was showed in Table 1. FTP grading was performed based on the visualization of structure in the mouth without protrusion of the tongue to evaluate the relative position of tongue to soft palate. The FTP was evaluated and graded as follows: grade I, the entire uvula, tonsils and pillars were clearly visible; grade II, the uvula was visible, while tonsils were invisible; grade III, only the soft palate was visible; grade IV, only the hard palate was visible (Friedman et al., 1999).

**TABLE 1:** Demographic and polysomnographic data.

	FTP I	FTP II	FTP III	FTP IV
Demographic data				
Gender	male	male	male	male
Age	35	48	51	38
BMI (kg/m^2^)	27.3	31.8	26.6	23.9
Neck circumference (cm)	43	44.5	42	37
Polysomnographic data				
AHI	28.2	67	46.4	18.5
Average SpO_2_ (%)	95.6	92.4	93.9	96.1
Lowest SpO_2_ (%)	85	75	79	87

Note: BMI, body mass index, which was calculated as weight/(height×height); AHI, apnea and hypopnea index, times/h; SpO_2_, oxygen saturation; FTP, Friedman tongue position.

### Ethical declaration

The Declaration of Helsinki was strictly followed. All participants signed the written informed consent form. The study was approved by the Ethics Committee of Beijing Tsinghua Changgung Hospital (Beijing, China; Approval No. 20150423-1). The study was also registered in the Chinese Clinical Trial Registry (Registration No. ChiCTR-OON-15006658).

### CT scans and reconstruction of a 3D airway model

CT images (LightSpeed Volume CT scanner; GE Healthcare Co., Ltd., Milwaukee, WI, United States) were acquired, and the CT scan parameters were as follows: tube voltage of 120 kV, tube current of 200–250 mAs, slice thickness of .625 mm, pitch of 3, resolution of 512 × 512 matrix. CT scan was performed under two conditions: MC and MO, using exactly all the same settings and the single lateral scout view. Subjects were relaxed, placed in a supine position, and should breathe smoothly *via* nose throughout CT scan. CT scan was first taken in the MC position. The head was positioned with the Frankfort plane (a line from infra-orbital rim to tragus of the ear) perpendicular to the bed. A lateral scout view was captured in a static MC position to plot the scanning scope from skull base to the level of five or six tracheal rings. To ensure the standardization and comparability, when CT scan was performed in the MO position, patients were instructed to open their mouth in a natural and comfortable state without any other body movement and to breathe smoothly *via* nose (Hu et al., 2018).

**FIGURE 1:**
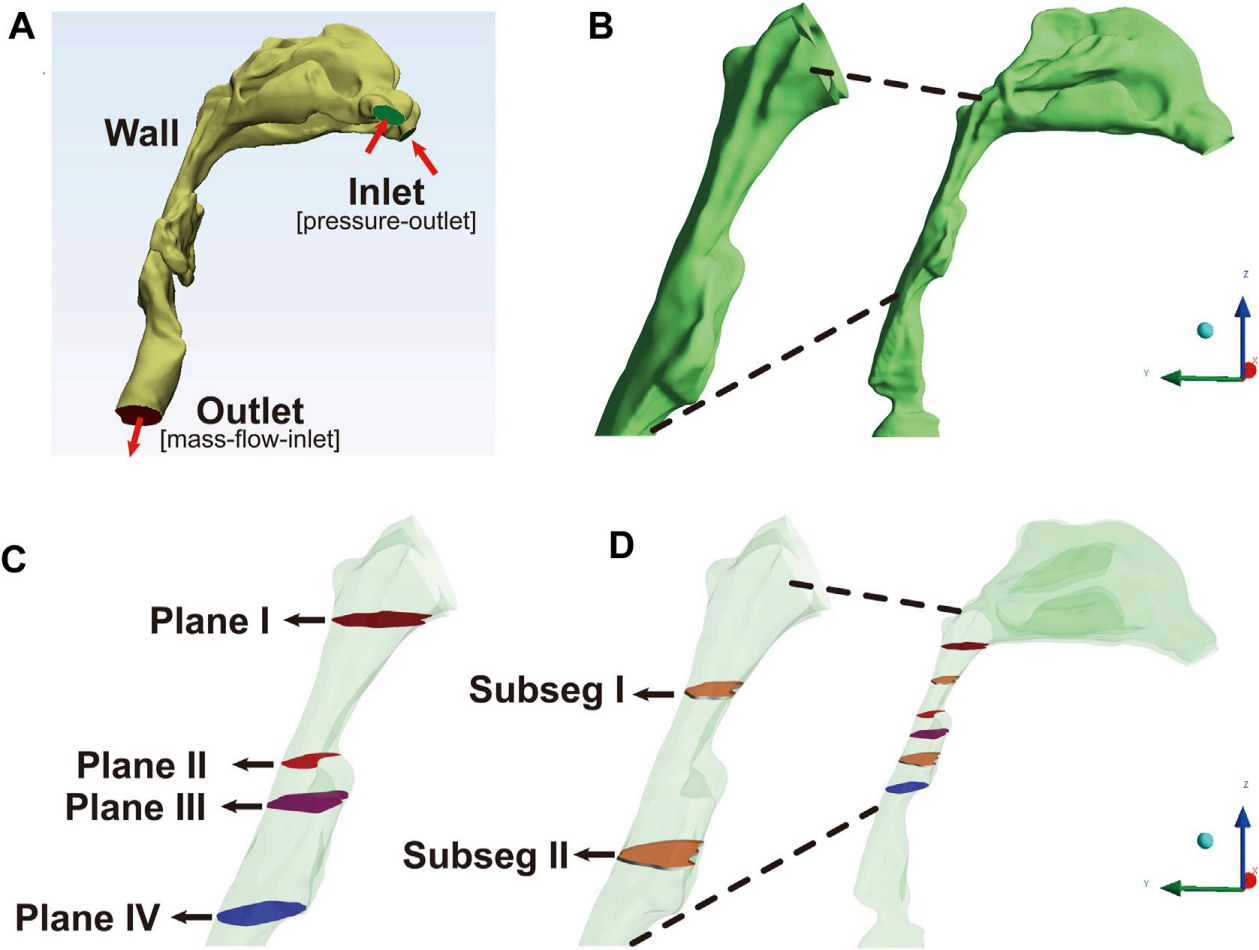
The reconstruction and segmentation of the airway model, Note: **(A)**, the anatomically accurate subject-specific airway model without sinus was constructed for numerical simulation. The inlet, corresponding to the anterior nostril, was set as pressure-outlet boundary. The outlet, corresponding to the level of five or six tracheal rings, was set as mass-flow-inlet boundary. **(B)**, the extracted model including velopharyngeal and oropharyngeal lumen as the regions of interest (ROIs) was used for post-processing analysis. **(C)**, Plane I, the first plane corresponding to the top of velopharyngeal lumen at the level of the hard palate; Plane II, the plane with the minimum area of the cross-sectional sections (plane _Amin_); Plane III, the plane corresponding to the interface between the velopharyngeal and oropharyngeal lumen; Plane IV, the lowest plane corresponding to the bottom of oropharyngeal cavity. **(D)**, subseg I and II represented the pharyngeal subsegments between plane N and plane N+1. Plane N was the arbitrary plane.

The image files were saved in Digital Imaging and Communications in Medicine (DICOM) format, and imported into the Mimics 17.0 software (Materialise, Leuven, Belgium) to process 3D reconstruction of the anatomically accurate subject-specific model. The upper airway was segmented by grouping voxels based on Hounsfield unit (HU) between upper threshold at 850 GV (gray value) and lower threshold at 0 GV into separate masks in each axial image. A 3D raw airway model, including the anatomy from anterior nostril to the level of five or six tracheal rings, excluding the paranasal sinuses, was reconstructed from these masks by surface triangulation (Figure 1A). Next, the raw airway model was exported into Geomagic Studio 12.0 software (Geomagic Inc., Cary, NC, United States). Some geometric singularities were corrected, and the decimation of polygonal surfaces and surface analysis were performed. Finally, a smoothing 3D model with NURB surfaces was established for meshing (Zhu et al., 2019).

### Grid generation and grid independence test

The smoothing airway model was then imported into the ICEM-CFD software (ANSYS Inc., Canonsburg, PA, United States) for further model repairing and mesh generation. The volume of the airway model was discretized using unstructured hybrid tetrahedral/hexahedral mesh elements. A tetrahedral mesh with several layers was generated inwards, starting from triangular surface mesh on airway wall. The inner core was filled with hexahedral elements (Mylavarapu et al., 2013).

The grid independence test was performed to determine the appropriate number of elements of a domain to make a compromise between the accuracy of numerical simulation and the computational cost (Liu et al., 2016; Faizal et al., 2020). Different grid sizes were selected to conduct the numerical simulation under the respiration with a flow rate of *Q* = 18 L/min. The velocity and pressure in the selected observation points of the reconstructed upper airway model of FTP I with MC were used as convergence criteria. When variations in the average values of velocity and pressure were <1%, an acceptable level of grid-independence was achieved, and the optimal number and size of the elements for CFD analysis were defined (Liu et al., 2016; Faizal et al., 2020). In the idealized grid size, .8–1.1 million elements were included. Then, the mesh was refined for several times to improve its quality and to ensure the maximum equiangular skew of cells within a range of .7–.8 in the present study.

### CFD simulation

The mesh was exported as standard tessellation language (STL) file, and then, it was imported into ANSYS Fluent 18.0 software (ANSYS Inc.) to solve the Reynolds-averaged Navier-Stokes (RANS) equations for the steady-state airflow simulation. The gravitational effect, heat transfer, phase change, and chemical reactions were all ignored. The flow was assumed to be steady-state, homogeneous, incompressible, adiabatic, and Newtonian. The continuity equation and the momentum equation were used to compute the velocity and the pressure of the flow. Airflow in the upper airway model was an unsteady transitional or turbulent flow with a relatively low Reynolds number (Xu et al., 2006; Liu et al., 2016). The Reynolds number at the pharyngeal constriction was estimated to be approximately 2–6 × 10^3^ (Mylavarapu et al., 2013). It was demonstrated that the steady-state RANS equations with the shear stress transport (SST) k–ω turbulence model using low Reynolds number corrections showed a better performance in predicting transitional turbulence flow/laminar-transitional-turbulent flow, which was the predominant flow characteristics in pharyngeal airway (Xu et al., 2006; Wen et al., 2008; Mylavarapu et al., 2013; Luo et al., 2014; Liu et al., 2016). The approach has been previously used to solve the flow field in several upper airway aerodynamics studies on patients with OSAHS (Mylavarapu et al., 2013; Luo et al., 2014) and its accuracy has been verified by experimental data of upper airway models (Xu et al., 2006; Wootton et al., 2014).

The semi-implicit method for pressure-linked equations (SIMPLE) algorithm was used for coupling the velocity and pressure fields (Wen et al., 2008; Mylavarapu et al., 2013; Zheng et al., 2017). The flow governing equations were discretized on the computational domain using second-order finite-volume schemes. During inspiration, an atmospheric stagnation pressure condition (pressure-outlet, total pressure = 0) was set at the nostril *inlet*. The negative value of a constant flow rate (mass-flow-inlet, *Q* = −18 L/min) with a uniform flow perpendicular to *outlet* was set at the bottom of the level of five or six tracheal rings (Figure 1A). Turbulence intensity was set to 5%. The hydraulic diameter at the *inlet* was set to 25 mm, and the *outlet* was set to 35 mm. A no-slip condition was imposed on the wall of upper airway. Convergence was determined by monitoring the magnitude of the absolute residual sources of mass and momentum. The number of iterations was set to 10^4^ steps, and the iteration continued until the residuals were below 1 × 10^−6^. Hybrid initialization was used to optimize the flow field.

### CFD post-processing

The results of CFD simulation were imported into CFD-Post software (ANSYS Inc.) and Tecplot software (Tecplot, Inc.) for the visualization and analysis of flow characteristics. Although the upper airway model from anterior nostril to the level of five or six tracheal rings was used for CFD simulation, the velopharyngeal and oropharyngeal airway segments as the regions of interest (ROIs) were eventually extracted for post-processing of data (Figures 1A, B). The first transversal cross-sectional plane at the level of the hard palate perpendicular to the *Z*-axis (Figure 1C, plane I) was identified as the top of velopharyngeal cavity. The descending cross-sectional planes (plane N, the arbitrary plane) parallel to plane I were generated downward per .5 mm along the *Z*-axis until the tip of epiglottis. The lowest cross-sectional plane was identified as the bottom of oropharyngeal cavity (Figure 1C, plane IV). The cross-sectional plane located at the level of the tip of soft palate was defined as the interface between the velopharyngeal and oropharyngeal airway segments (Figure 1C, plane III). The plane_Amin_, defined as the plane with the minimum area of the cross-sectional planes, should have a smaller area than both the upper and lower planes (Figure 1C, plane II). The ROIs were divided into several small subsegmental lumens (subseg N, Figure 1D) with the top as plane N, the bottom as plane N+1, and the pharyngeal wall between plane N and plane N+1 (plane N was the arbitrary plane).

The area, the mass flow-weighted average (massFlowAve) of static pressure (*Ps*), velocity (*v*), and TKE in each cross-sectional plane were calculated. The magnitude of WSS_subseg N_ on each subsegmental wall was acquired. In addition, the area ratio of plane N to plane_Amin_ (Eq. 1) was calculated to explore the variation tendency of pharyngeal lumen.

$$Area\;ratio=\frac{{Area}_{plane\;N}}{{Area}_{plane\;N+1}}$$ (1)

The massFlowAve of dynamic pressure (*Pv*) and the total pressure (*Pt*) in each cross-sectional plane were calculated using the following equations (Legg, 2017):

Pt=Pv+Ps (2)

$$Pv=\frac{1}{2}*\;\rho \;*\;v^{2}$$ (3)

The resistance in each sequential subsegmental lumen (*R*_subseg N_) was calculated independently to determine the variation tendency of resistance in the subsegmental lumens along the length of ROIs.

## Results

### Morphological changes from MC to MO

**FIGURE 2:**
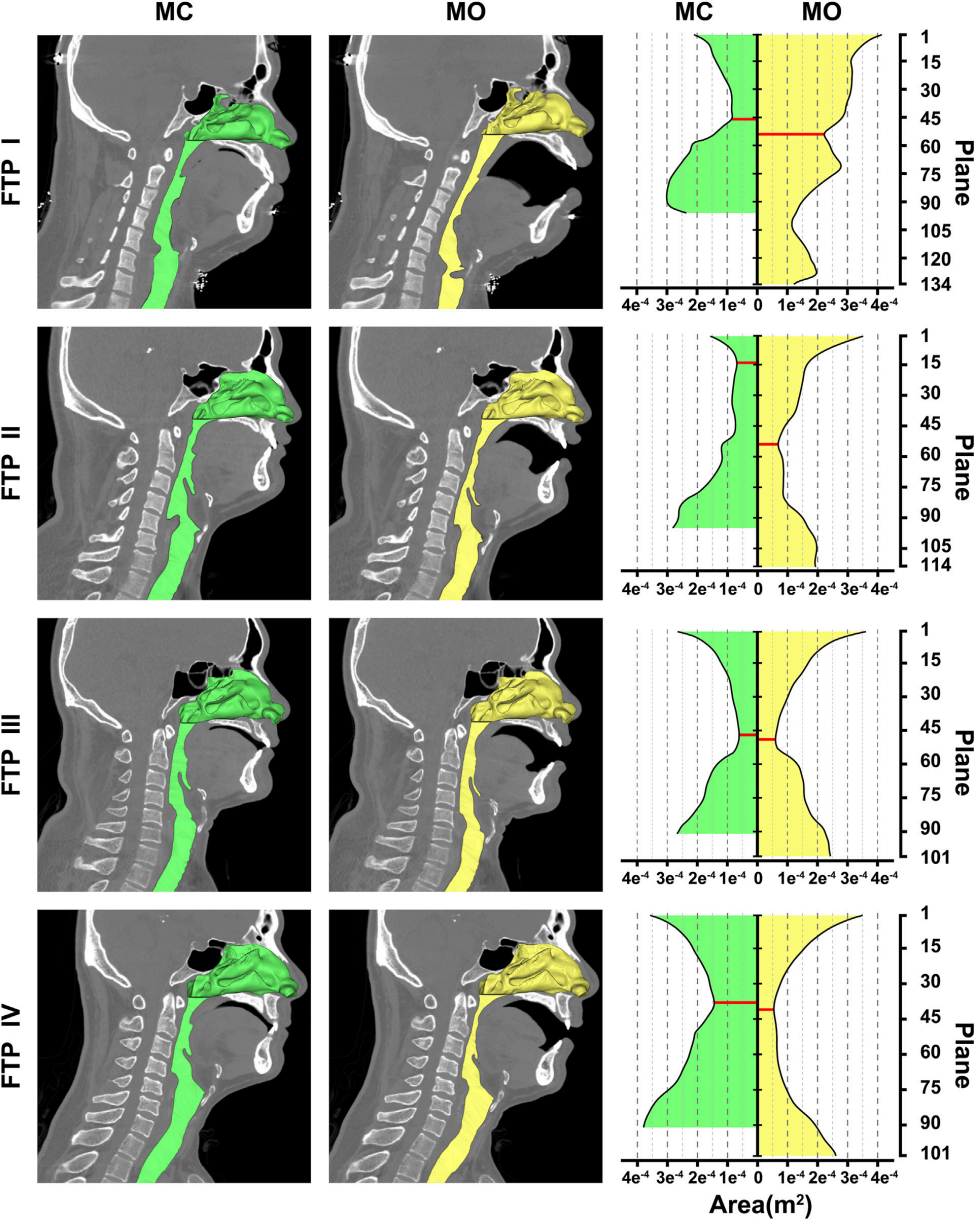
Morphological changes of the airway between MC and MO, Note, the airway models were embedded on the median sagittal CT image in the left two columns. The nasal cavity and nasopharynx presented in the form of stereoscopic (three-dimensional, 3D) and the remaining airway presented with sagittal profile. The green color represented the baseline airway lumen and the yellow color represented the airway lumen after MO. The area contours of cross-sectional planes from the level of the hard palate to the tip of epiglottis were showed in the right column. The horizontal axis represented the area of planes (m^2^); the longitudinal axis represented the plane N. The red solid line represented the plane_Amin_. (MC, mouth closed position; MO, mouth opening position; FTP, Friedman Tongue Position).

As illustrated in Figure 2, the green color on the mid-sagittal CT image represented the baseline upper airway lumen and the yellow color represented the upper airway lumen after MO. The ROIs were lengthened in the four subjects from MC to MO (Table 2; Figure 2). The area of velopharyngeal plane_Amin_ enlarged from MC to MO for FTP I (from .8288 to 2.226 cm^2^), and decreased from baseline to MO for FTP IV (from 1.4440 to .5443 cm^2^). MO also led to a change in the location of plane_Amin_ of the ROIs. MO resulted in the lowering of the plane_Amin_, which was originally located at the velopharyngeal level (plane_44_), to the oropharyngeal level (plane_102_) for FTP I. Moreover, MO caused a variation in the level of plane_Amin_ from a baseline position close to the top of velopharyngeal cavity (plane_14_) to the vicinity of the tip of soft palate (plane_54_) for FTP II.

**TABLE 2:** Morphological characteristics of the ROIs between MO and MC based on FTP rating.

	FTP I		FTP II		FTP III		FTP IV	
	MC	MO	MC	MO	MC	MO	MC	MO
Extracted pharyngeal segment								
Length (cm)	4.75	6.65	4.72	5.65	4.52	5.01	4.53	5.12
The sum total of planes	96	134	95	114	91	101	91	103
Dividing plane (plane No.)	58th	70th	54th	68th	68th	68th	51st	53rd
Velopharyngeal cavity								
Plane_Amin_ (plane No.)	44th	54th	14th	54th	47th	49th	38th	41st
The area of Plane_Amin_ (cm^2^)	.8288	2.226	.6883	.6862	.6074	.6035	1.4440	.5443
Oropharyngeal cavity								
Plane_Amin_ (plane No.)	59th	102nd	61st	77th	69th	69th	52nd	54th
The area of Plane_Amin_ (cm^2^)	2.037	1.153	1.177	.8473	1.649	1.533	2.085	.6441

Note: ROIs referred to the extracted velopharyngeal and oropharyngeal segment. The dividing plane was defined as the interface between the velopharyngeal and oropharyngeal airway. The plane_Amin_, defined as the minimum area of the cross-sectional plane, should have smaller area than both the upper and lower plane. More detailed description of the localization methods and definition of the planes were presented in Method Section. (MC, mouth closed position; MO, mouth opening position; FTP, Friedman Tongue Position).

### The trend of pressure changes from MC to MO

**FIGURE 3:**
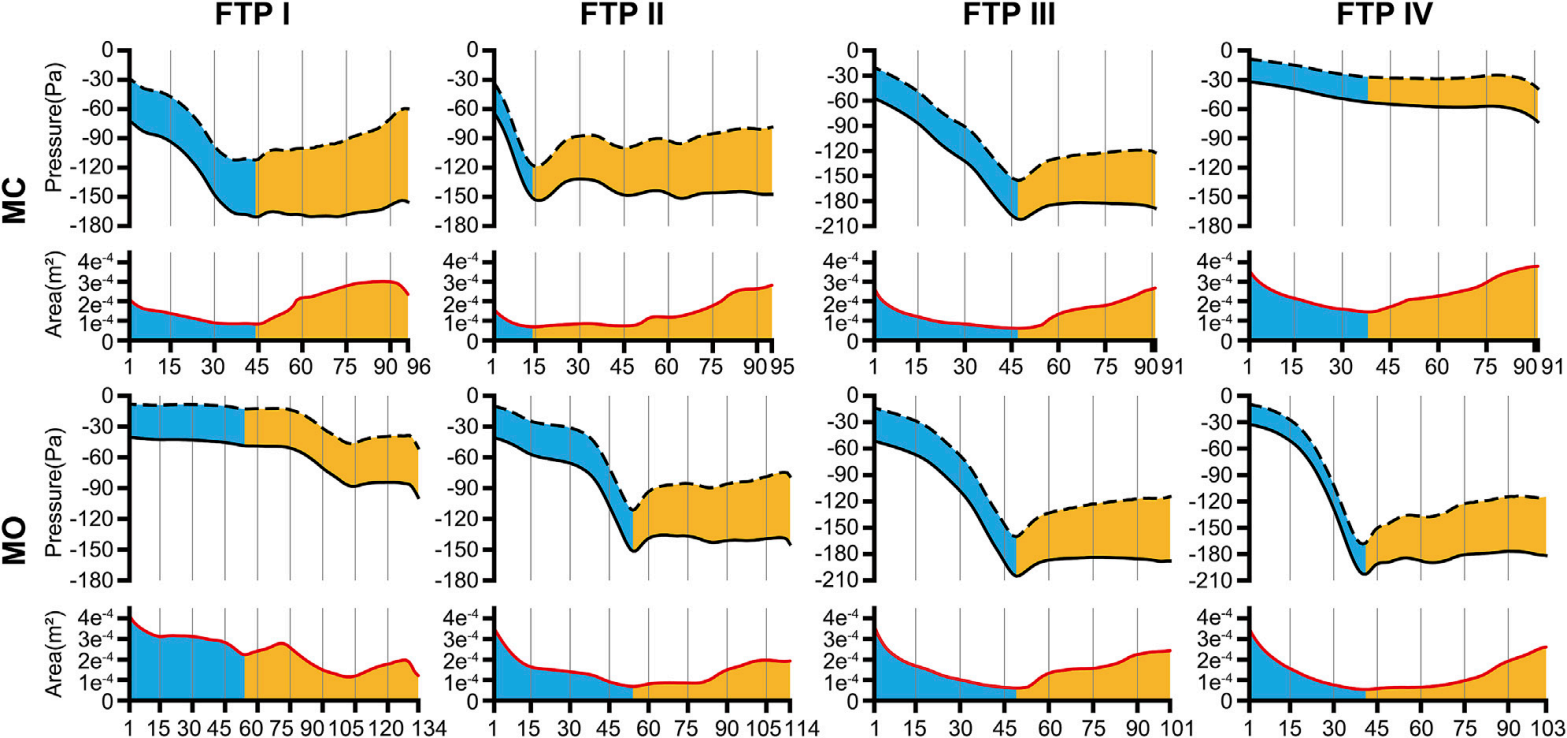
The tendency of pressure changes along with the area change from MC to MO. Note, the trend of pressure changes was showed in the upper part of each line graph. The black dotted line was the negative value of dynamic pressure (−*Pv*) and the black solid line was the static pressure (*Ps*). The value of the gap between black solid line and black dotted line could be considered as the total pressure (*Pt*) according to Eq. 4. The trend of area changes was showed in the lower part of each line graph. The red solid line was the area of cross-sectional planes. The horizontal axis represented the plane N. The cutoff plane in each line graph represented the velopharyngeal plane_Amin_. (MC, mouth closed position; MO, mouth opening position; FTP, Friedman Tongue Position).

In the inspiratory phase, the negative pressure gradient results from the contraction of pleural pressure drives the airflow with a direction from the nostril to pulmonary alveoli. Relative to a datum of atmospheric pressure, the *Ps* value was negative, while the *Pv* value, which was calculated by Eq. 3, was always positive. The variation trends of *Ps* (the black solid line) and the negative value of *Pv* (the black dotted line) are shown in Figure 3. According to Eq. 4, the gap between the black solid line and the black dotted line in Figure 3 should be equal to the *Pt* value.

$$P_{gap}=Ps-\left(-Pv\right)=Ps+Pv=Pt$$ (4)

**FIGURE 4:**
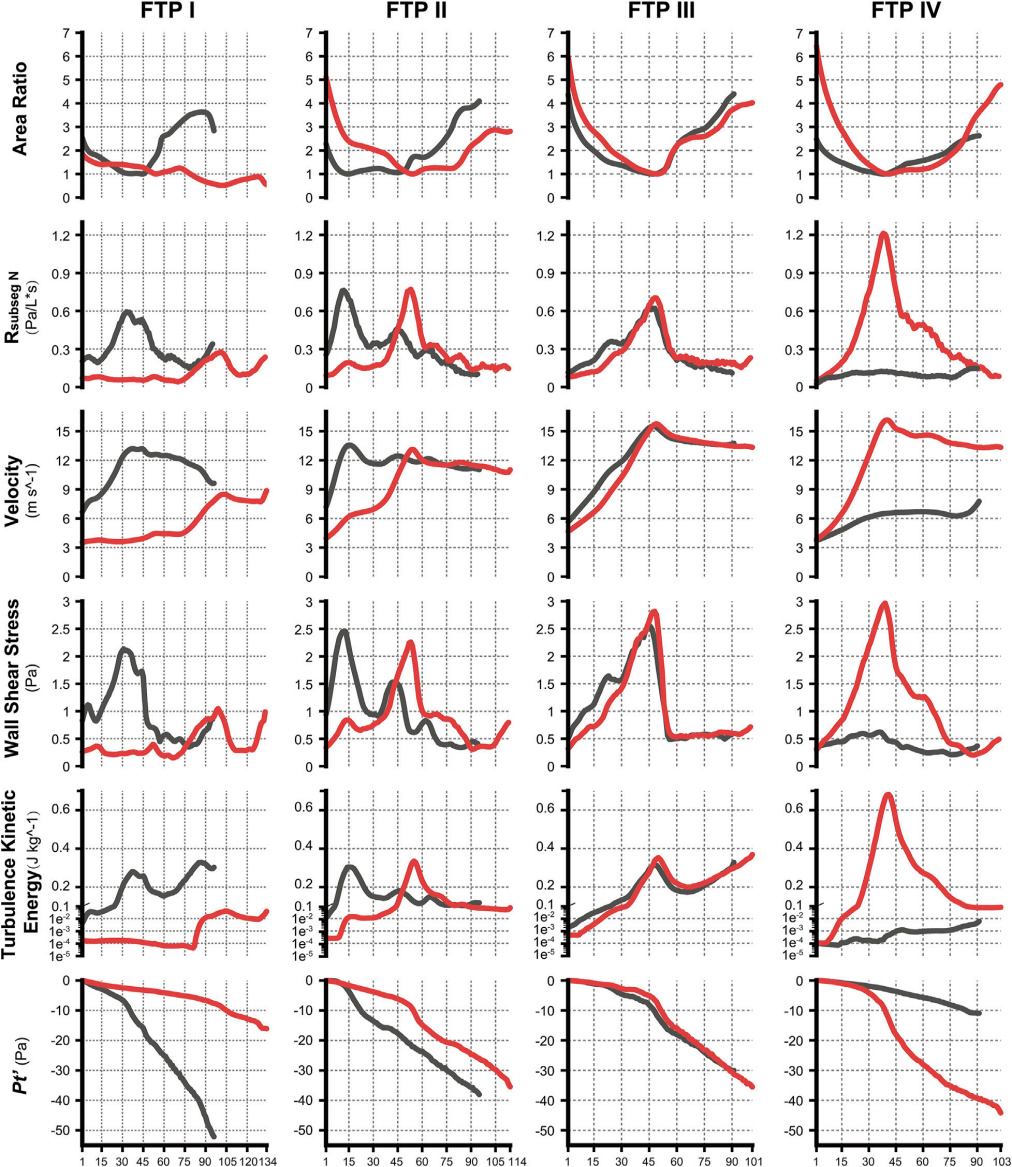
The variation tendency of CFD variables between MC and MO. Note, the black lines represented values of CFD variables in MC while red lines represented values in MO. The horizontal axis represented the plane N or subseg N. (CFD, computational fluid dynamics; MC, mouth closed position; MO, mouth opening position; FTP, Friedman Tongue Position).

As illustrated in Figure 3, the pressure distribution in the ROIs for FTP I, II, III with MC and FTP II, III, IV with MO followed a similar trend. That was, with a gradual narrowing lumen due to the arc-like dorsal surface of soft palate, the *Ps* value decreased gradually along with the increase of the *Pv* value. Then, the *Ps* reached its maximum negative value, and the *Pv* yielded its maximum value at the plane_Amin_. In the downstream plane_Amin_, it was the progressive expansion in the remaining pharyngeal lumen that there would be a rise in the *Ps* value along with a drop in the *Pv* value. It is noteworthy that, for FTP I with MO, the *Pv* and *Ps* values showed a more stable trend in the expanded velopharyngeal lumen after MO, and a decrease of *Ps* value to its minimum value at the new plane_Amin_ in the relatively constricted oropharyngeal lumen. For FTP IV with MC, the stable tendency of the *Pv* and *Ps* values depended on the minor variation in the cross-sectional area of overall ROIs (Figure 4).

In the present study, the total pressure at the *inlet* (pressure-outlet) was set to zero whilst a fixed flow rate was specified at the *outlet* (Figure 1A). The continuity of flow and pressure distribution determined that the pressure magnitude at each plane of the ROIs (Figure 3) should include the pressure reduction in nasal cavity and nasopharynx. Thus, a new variable *Pt*’ was introduced, as formulated in Eq. 5, to eliminate the geometric influences of upstream airway and to analyze the variations of the total pressure drop in the ROIs associated with MO.

$$Pt{’}_{plane\;n}=Pt_{plane\;n}-Pt_{plane\;I}$$ (5)

The variations of *Pt’*_plane n_ are displayed in Figure 4.

### The changes in velocity contours and TKE from MC to MO

**FIGURE 5:**
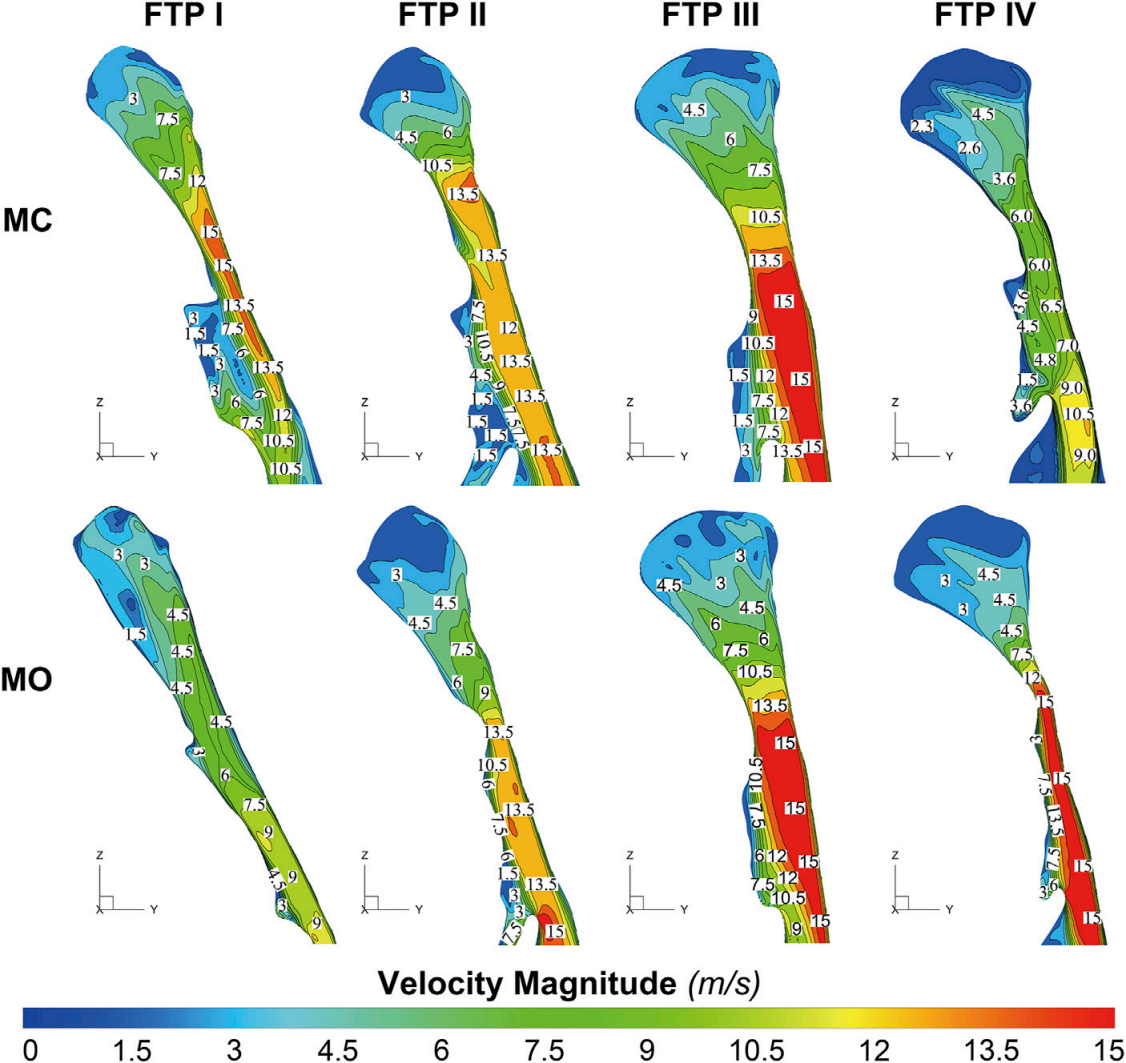
The contour of velocity magnitude on the median sagittal plane. Note, MC, mouth closed position; MO, mouth opening position; FTP, Friedman Tongue Position.

**FIGURE 7:**
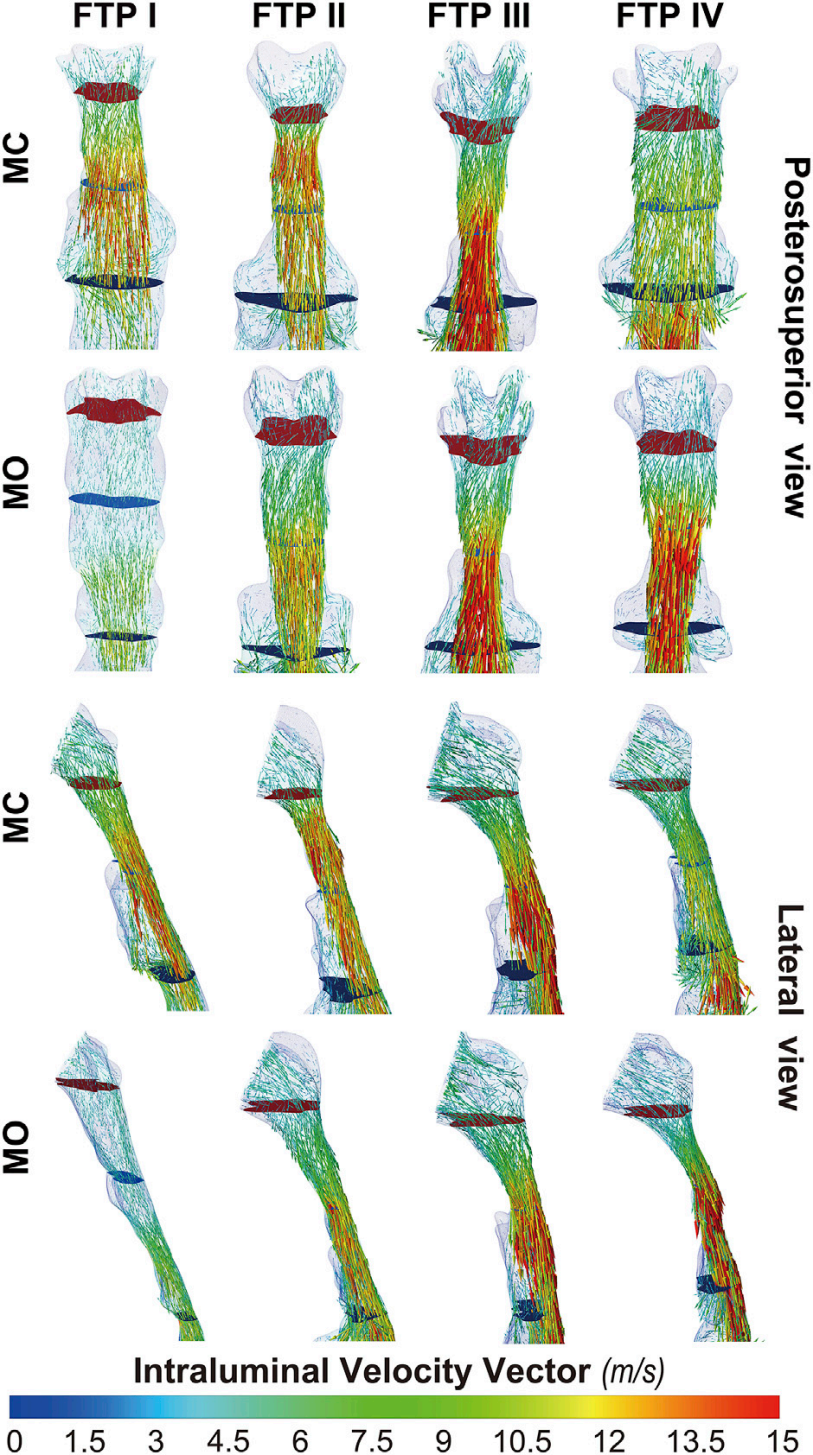
The intraluminal velocity vector plot of pharyngeal cavity. Note, the uppermost plane with dark red color was defined as the top of velopharyngeal lumen. The lowest plane with green color was defined as the bottom of oropharyngeal lumen. (Sampling, vertex; Max points, 5,000; MC, mouth closed position; MO, mouth opening position; FTP, Friedman Tongue Position).

As illustrated in Figure 4, the velocity and TKE of airflow gradually increased with the pharyngeal constriction. It could be observed from the contours of velocity in mid-sagittal plane (Figure 5) that a mainstream flow strip with high velocity and TKE termed “pharyngeal jet” began to form as flow was passing through the constriction (Allen et al., 2004; Xu et al., 2006; Jeong et al., 2007; Persak et al., 2011; Powell et al., 2011; Sul et al., 2014; Liu et al., 2016; Faizal et al., 2020; Feng et al., 2021). For FTP I with MC, a jet with increasing TKE was formed by the velopharyngeal constriction and it extended along the posterior pharyngeal wall into oropharyngeal lumen (Figures 4–7). For FTP I with MO, at the same flow rate, more stable airflow passed through the velopharynx into the upper oropharynx due to the luminal expansion associated with MO (Figures 4–7). The jet with relative lower velocity and TKE began to form approximately at the plane_Amin_ of the oropharyngeal lumen. For FTP II, the initial site of the baseline pharyngeal jet with high velocity and TKE corresponding to the top of the velopharynx moved inferiorly to the vicinity of the bottom of velopharynx after MO (Figures 4, 5). For FTP III, the profiles of pharyngeal jet, velocity contours, and magnitude of TKE did not change significantly due to the insignificant geometric difference from MC to MO. The appearance of velocity contours and pharyngeal jet for FTP IV with MC resembled that for FTP I with MO. However, for FTP IV with MO, a stronger pharyngeal jet with the highest magnitude of velocity and TKE occurred at the remarkable velopharyngeal constriction and even extended from the velopharynx into the hypopharynx (Figures 4–7).

Downstream the plane_Amin_, for FTP I with MC, the rapid luminal expansion due to the curvature of tongue dorsum caused the anterior flow separation with recirculation zones formation where the TKE decreased transiently and increased again. Similar phenomenon could also be found for FTP III regardless of MC and MO. For FTP II, irrespective of MC (planes 14–54) and MO (planes 54–79), a long restricted pharyngeal segment could be observed downstream the plane_Amin_, and the slightly expanded luminal diameters might subsequently contribute to the weaker flow separation and the smaller recirculation zones corresponding to the lower level of TKE. For FTP IV with MO, downstream the plane_Amin_, the relatively smaller recirculation zones corresponding to the relatively narrower oropharyngeal lumen, which presented with less obvious expansion, predicted the continuous decrease of TKE (Figures 4–7).

### The changes in WSS from MC to MO

**FIGURE 8:**
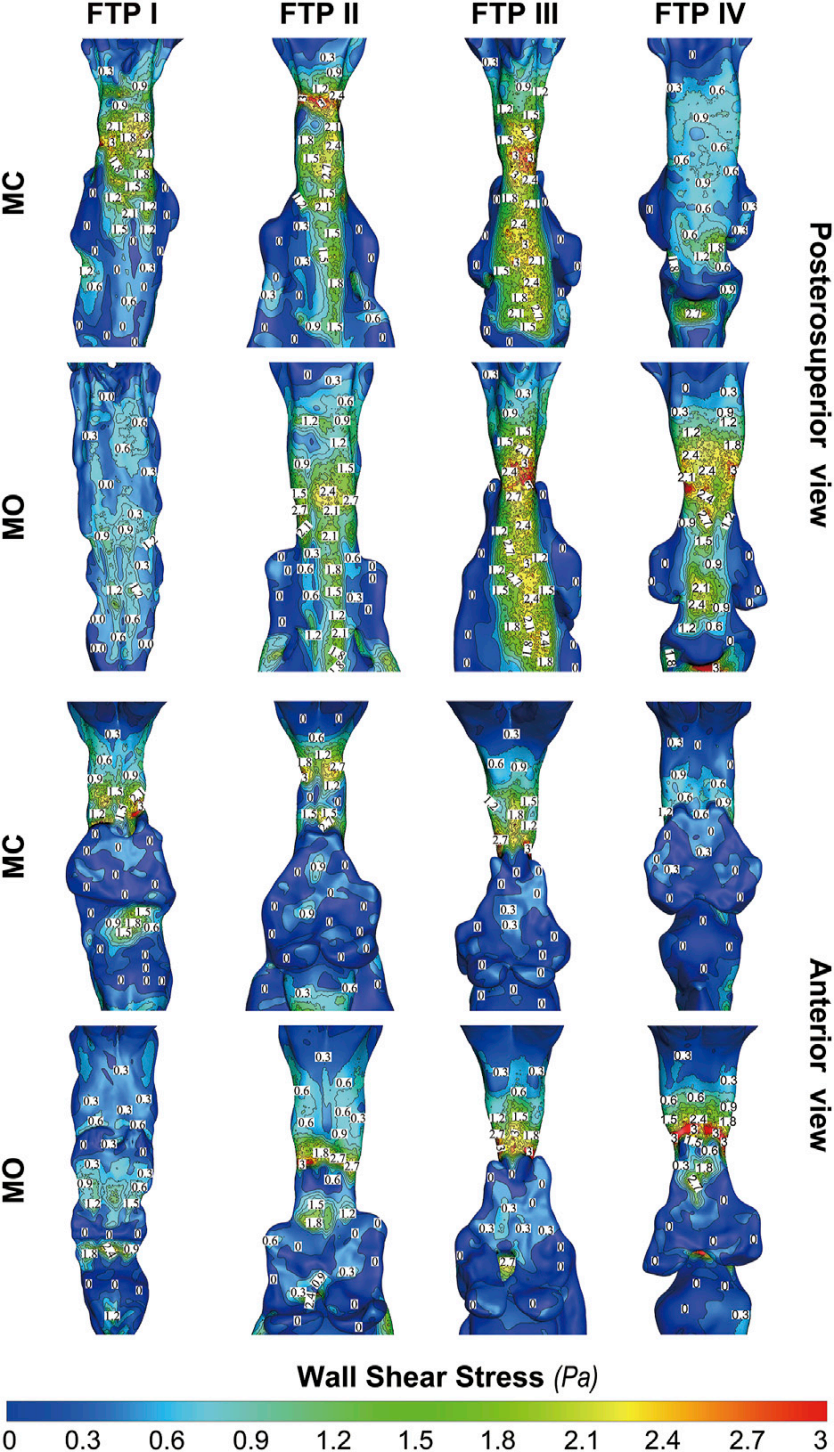
The contour of wall shear on the pharyngeal wall. Note, MC, mouth closed position; MO, mouth opening position; FTP, Friedman Tongue Position.

Figure 8 shows the WSS distribution on the pharyngeal wall. The WSS distribution was generally presented as an indicator of the interaction between flow and pharyngeal wall. For FTP I with MC, the magnitude of WSS increased with the progressive narrowing pharyngeal lumen above the plane_Amin_ (Figures 4, 8). The plane_Amin_ corresponded to the highest values of velocity and WSS (red color, Figures 4, 8). As mentioned above, a pharyngeal jet resulted from the velopharyngeal constriction flowed downstream and impinged on the posterior pharyngeal wall that corresponded to the WSS distribution (Figures 5, 7, 8). Additionally, after the plane_Amin_, the flow separation and recirculation occurred due to the downstream airway expansion. As illustrated in Figures 7, 8, a significantly lower (or near zero) WSS could be observed in the anterior and lateral oropharyngeal wall because the downstream separation flow moved in the upstream direction in the anterior oropharyngeal cavity that corresponded to the recirculation regions. For FTP I with MO, the variations of WSS were similar to those of velocity contours, in which a relatively higher WSS could be observed in the lower part of oropharyngeal cavity (Figures 5, 7, 8). For FTP II, the maximum WSS was observed in the region close to the top of velopharyngeal lumen for MC, while for MO, it was found in the region close to the bottom of velopharyngeal lumen (Figure 8). For FTP III, a similar tendency of WSS distribution was observed in both MC and MO. For FTP IV with MO, a longer vigorous jet-like flow with a higher velocity and a stronger jet impingement on the posterior pharyngeal wall contributed to the elevated WSS (Figures 4, 5, 7, 8).

### The changes in resistance from MC to MO

Airway resistance is determined by the Hagen-Poiseuille equation which measures the pressure difference across the airway and divides the difference by the flow. This equation highlights key point related to resistance under laminar flow conditions (Kaminsky, 2012). However, laminar, unsteady transitional and turbulent flow could be found in human upper airway that could result in a mathematically complex relationship between airway geometry and flow (Xu et al., 2006; Wen et al., 2008; Kaminsky, 2012; Mylavarapu et al., 2013; Luo et al., 2014; Liu et al., 2016). When flow is turbulent, the Hagen-Poiseuille equation should be modified (Kaminsky, 2012). According to the “Fluid Flow: General Principles,” it was assumed that the friction and the pressure loss coefficients (*K*) of a short subsegment (subseg N) remained constant in the steady-state airflow simulation, that’s, they were both independent of the Reynolds number (Legg, 2017). The resistance of each subsegmental lumen (*R*_subsge n_) could be defined by the *square law* (Kaminsky, 2012; Legg, 2017), namely Eq. 6 (*V*, volumetric flow rate):

$$\Delta Pt=R\;*\;V^{2}$$ (6)

ΔPt=K * Pv (7)

$$R=K*\frac{Pv}{V^{2}}$$ (8)

According to Eqs 6–8, the following equations were derived: (*ρ*, gas density; A, area)

$$R_{subseg\;N}=K_{subseg\;N}*\frac{Pv_{plane\;N}}{V_{plane\;N}^{2}}$$

$$R_{subseg\;N}=K_{subseg\;N}*\frac{\frac{1}{2}*\rho *v_{plane\;N}^{2}}{{\left(v_{plane\;N}*\frac{A_{plane\;N}+A_{plane\;N+1}}{2}\right)}^{2}}$$

$$R_{subseg\;N}=K_{subseg\;N}*\frac{\frac{1}{2}*\rho }{{\left(\frac{A_{plane\;N}+A_{plane\;N+1}}{2}\right)}^{2}}$$

$$R_{subseg\;N}=\frac{Pt_{plane\;N}-Pt_{plane\;N+1}}{Pv_{plane\;N}}*\frac{\frac{1}{2}*\rho }{{\left(\frac{A_{plane\;N}+A_{plane\;N+1}}{2}\right)}^{2}}$$

$$R_{subseg\;N}=\frac{Pt_{plane\;N}-Pt_{plane\;N+1}}{\frac{1}{2}*\rho *v_{plane\;N}^{2}}*\frac{\frac{1}{2}*\rho }{{\left(\frac{A_{plane\;N}+A_{plane\;N+1}}{2}\right)}^{2}}$$

$$R_{subseg\;N}=\frac{Pt_{plane\;N}-Pt_{plane\;N+1}}{{\left[v_{plane\;N}*\left(\frac{A_{plane\;N}+A_{plane\;N+1}}{2}\right)\right]}^{2}}$$ (9)

According to Eq. 9, resistance in the subsegmental lumen (R_subseg N_) was directly related to the ratio of the total pressure loss (Δ*Pt*_subseg N_) to the *Pv*_plane N_, inversely to the square of the mean value of A_plane N_ and A_plane N+1_. As shown in Figure 4, for FTP I, II, III with MC and for FTP II, III, IV with MO, the resistance increased along with the constrictive lumen above the level of plane_Amin_. The magnitude of resistance reached its maximum at the level of plane_Amin_. Additionally, the smaller the area of plane_Amin_, the higher the magnitude of resistance. For FTP IV, MO led to a smaller area of plane_Amin_ corresponding to a 1.5-fold higher magnitude of resistance (A_plane 41_ = .5443 cm^2^; R_subseg 40_ = 1.27 Pa) than that for FTP II (A_plane 54_ = .6862 cm^2^; R_subseg 53_ = .8271 Pa) and FTP III (A_plane 49_ = .6035 cm^2^; R_subseg 48_ = .7624 Pa). Downstream the plane_Amin_, the resistance in subsegmental lumen gradually decreased with the gradual expansion of pharyngeal lumen (Figure 4).

## Discussion

MO induced a different tendency in morphological changes of velopharyngeal and/or oropharyngeal lumens in subjects with different phenotypes of FTP grades (Figure 2; Table 2). According to the definition of FTP, the higher the FTP grade, the more overlap between tongue and soft palate (Friedman et al., 1999). For FTP I, the backward movement of tongue base with MO was responsible for the narrower oropharyngeal lumen, while it had less impact on soft palate owing to the smallest overlap. A soft palate could be viewed as a “curtain” attaching to the posterior end of the hard palate. The inferior movement of tongue with MO stretched palatoglossal arch, which connected the soft palate to the tongue base. It could stiffen the soft palate and improve the velopharyngeal constriction (Hu et al., 2018). For FTP II and III, the tongue base retraction resulted from MO was partially counteracted by the supporting force arising from the overlapping portions of the soft palate (Hu et al., 2018). For FTP IV, the tongue and the soft palate had the most overlapping region and presented with anterior-posterior locations in an upright position and dorsal-ventral locations in a supine position (Hu et al., 2018). Given that the root of soft palate anchored to the hard palate and the tip of soft palate was in the relatively free state, the closer to the tip of soft palate, the greater the amplitude of soft palate retraction due to the posterior displacement of tongue associated with MO, and eventually, the sharper the tendency of the velopharyngeal constriction (Figure 4, area ratio).

As shown in the contours of cross-sectional area (Figure 2), the ROIs from the hard palate to the tip of epiglottis were analogous to an hourglass. It always had a constrictive “neck” corresponding to the plane_Amin_ (FTP I with MC; FTP III with MC and MO) or a certain length of constrictive segment (FTP II with MC and MO, FTP IV with MO) that was commonly located at, while was not necessarily confined to (FTP I with MO), the velopharyngeal region. The hourglass-shaped ROIs also had two individual opposing lumen tapers. As displayed in Figures 3, 4, the inverted-cone-shaped pharyngeal segment above the constrictive “neck” presented with a gradual narrowing lumen that resulted in gradually increased airway resistance to flow. Subsequently, a depression *Ps* was required to drive the airflow with an increased velocity (an increase in *Pv* value) to maintain the specified flow rate (FTP I with MC; FTP II, FTP III, and FTP IV with MO). The remaining pharyngeal segment, which was progressive expansion to varying degrees from the so-called constrictive “neck” to the tip of epiglottis, appeared as a pear. For FTP I with MC, as well as FTP II, FTP III, and FTP IV with MO, the resistance gradually decrease with the gradual expansion of pharyngeal lumen, and there was subsequently a rise in *Ps* value (*Ps* regain) accompanied by a drop in *Pv* value (a decrease in velocity) (Figures 3, 4). However, confusion arose that although a relatively less resistance in the expansive segment might require a lower negative *Ps* value to drive the airflow, the absolute value of *Pt*’ (Figure 4, the gap between *Ps* and −*Pv* in Figure 3), an indicator that can be considered as a surrogate for energy loss (Bates et al., 2016b; Legg, 2017), continued to increase within the expansive segment. It is essential to indicate why this phenomenon occurred?

**FIGURE 9:**
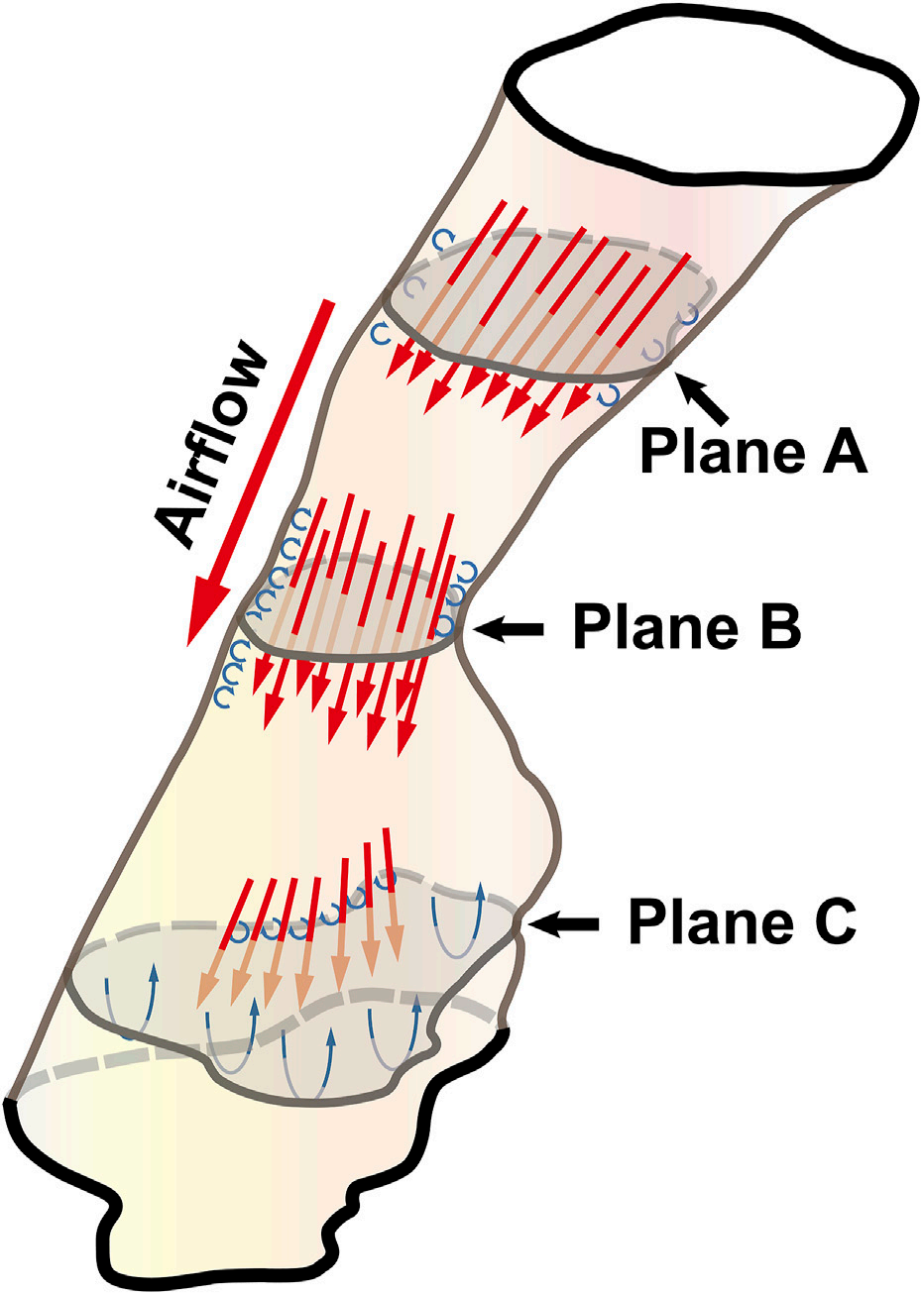
The illustration of the pharyngeal flow during inspiration. Note, Plane A was a random cross-sectional plane of the velopharyngeal cavity. Plane B corresponded to the pharyngeal plane_Amin_. Plane C was a random cross-sectional plane of the oropharyngeal cavity. The flow tended to be converged when it passed through Plane A to Plane B. The accelerated flow caused turbulence in the near-wall region that disrupted the boundary layer and resulted in the formation of small and fast rotating vortical structures (the small blue circulation). Downstream of plane B, luminal expansion led to flow separation and the formation of low-velocity flow recirculation region (the large blue circulation) where flow toward the upstream direction.

**FIGURE 6:**
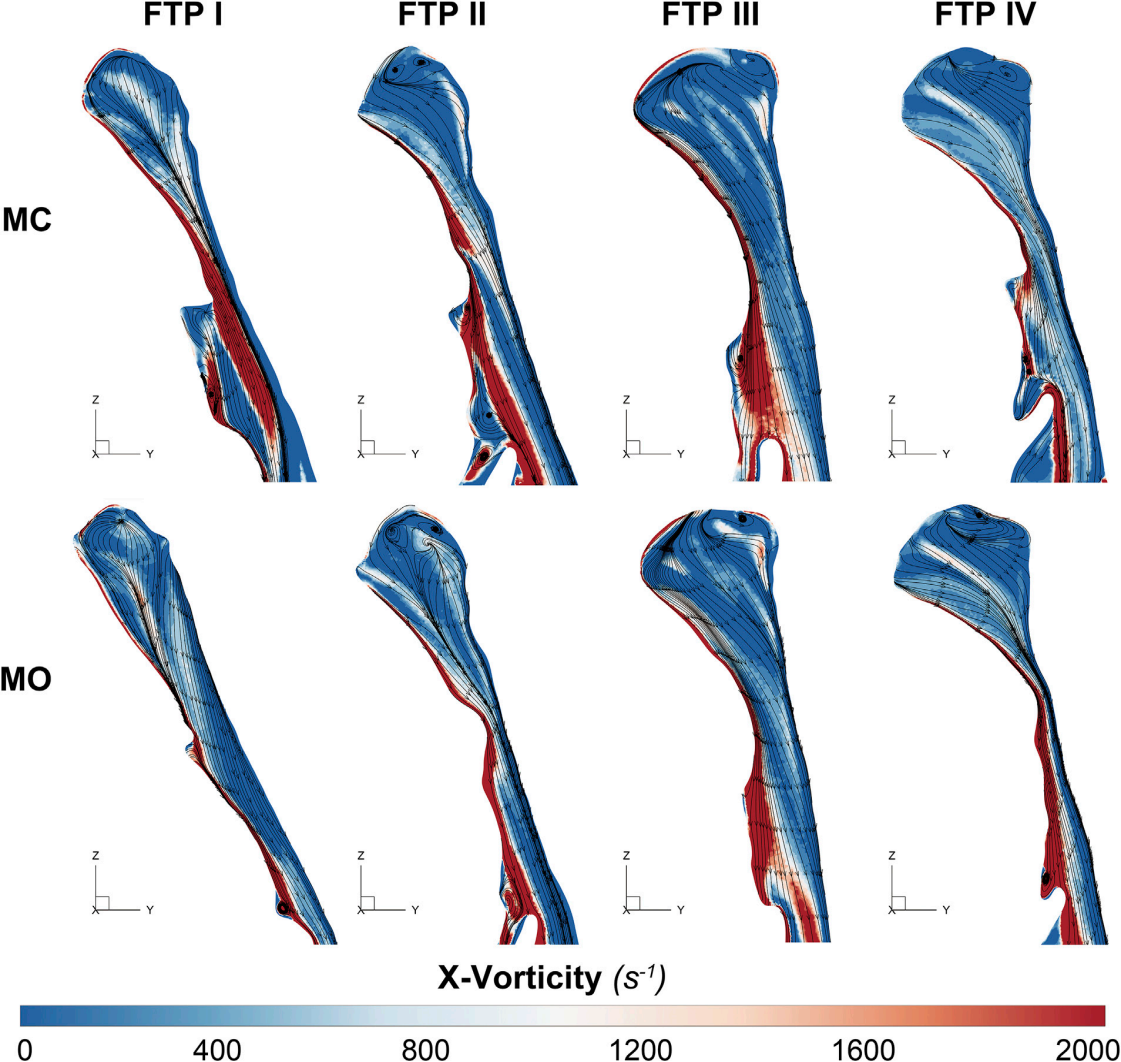
The contour of vorticity on the median sagittal plane. Note, Downstream the plane_Amin_, the vorticity is caused by the changes of regional flow direction in relation to the interface between the jet with a higher airflow velocity (located at the posterior and central part of lumen) and the recirculation region with a relatively lower airflow velocity (located at the anterior and lateral part of lumen). MC, mouth closed position; MO, mouth opening position; FTP, Friedman Tongue Position.

Governed by the Bernoulli equation, without considering the potential energies, the total energy of flow can be expressed in terms of *Ps* and *Pv* (Legg, 2017). Under an ideal state, the *Ps* of inward airflow may fall below atmospheric pressure to a value equal to the *Pv*, thereby maintaining zero *Pt* value (equal to *Ps* plus *Pv*, Eq. 2). Practically, the absolute value of *Ps* may be higher than that of *Pv* by an amount equal to the net pressure loss (Legg, 2017). Two components of pressure loss (a surrogate for energy loss) should be mentioned: frictional loss and interior flow loss (Bates et al., 2016a; Bates et al., 2016b). Frictional loss was mainly referred to the local resistive effect of the walls, and its magnitude could be quantified by WSS (Bates et al., 2016b). Airflow which accelerated in pharyngeal lumen led to turbulence, which disrupted the boundary layer and resulted in WSS (Qi et al., 2014). The WSS not only indicated the tangential drag force produced by airflow across the wall surface, but also represented the reaction force exerted by the inner luminal wall on the flow (Mihaescu et al., 2011; Bates et al., 2016b). Mihaescu et al. found that shear stresses were imposed on the wall as the jet impingement with the formation of small and fast rotating vortical structures (300–600 Hz) in the shear layers (Mihaescu et al., 2011) (Figure 9). The large number of small-scale eddies might result from jet impingement, which, in turn, might promote the jet dissipation related to WSS and increase the frictional loss (Mihaescu et al., 2011; Bates et al., 2016b; Taherian et al., 2019; Faizal et al., 2020). Downstream the constrictive region, luminal expansion due to the curvature of tongue dorsum caused flow separation, and formation of recirculation region (Mihaescu et al., 2011; Mylavarapu et al., 2013; Bates et al., 2016b) (Figures 5, 7, 9; FTP I with MC, FTP II, FTPIII, and FTP IV with MO). Interior flow losses were attributed to the flow that was not in equilibrium, including internal rearrangement of the flow (separation), unsteadiness (fluctuations in jet orientation), the dissipation of fluctuations, and turbulence (Bates et al., 2016a; Bates et al., 2016b). Professor Mihai Mihaescu indicated a larger and slower rotating recirculation bubble (20–40 Hz) would develop in the reversed flow region (Mihaescu et al., 2011) (Figures 7, 9). Due to the relatively lower velocity, the local frictional loss might be low or nearly zero corresponding to the low or nearly zero WSS that imposed on the anterior and lateral wall of recirculation region (Bates et al., 2016b) (Figures 7, 8). The interface between the jet with a higher airflow velocity (located at the posterior and central part of lumen) and the recirculation region with a relatively lower airflow velocity (located at the anterior and lateral part of lumen) might predict turbulent dissipation, jet breakdown and the elevated TKE, contributing to regionally higher interior flow loss (Mihaescu et al., 2011; Bates et al., 2016a; Bates et al., 2016b) (Figures 6, 7, 9).

The pharyngeal jet was the most typical aerodynamic feature that could be considered as the delivery carrier of kinetic energy. According to the formation, development, and breakdown of pharyngeal jet and flow separation, we attempted to separate the ROIs into three levels: level 1), the formation of jet; level 2), pre flow full separation; level 3), flow full separation. Level 1 could also be considered as the accumulation of kinetic energy of jet flow. The constant drop in negative static pressure (*Ps*) was converted into kinetic energy of flow (*Pv*) to overcome the increased airway resistance due to the gradual contraction of the lumen (Legg, 2017) (Figure 3). The *Ps* approached to its minimum value at the location corresponding to plane_Amin_ where the resistance, *Pv*, velocity, and TKE reached their maximum values and the pharyngeal jet was eventually formed (Figures 3–5). Simultaneously, the tangential shear forces exerting to the inner wall gradually increased and approached to its maximum value at the location of plane_Amin_ (Figures 3–5, 8). As reported by Bates et al. (2016b), the total pressure loss and frictional loss were nearly identical where the flow was well distributed (more convergent airflow in the process of jet formation, Figures 6, 9). The variation of *Pt’* in this level could be mainly due to the WSS-associated frictional loss (Figure 8). In level 2, there was a long or a short constant ring-shaped constrictive segment with a slight luminal expansion downstream the plane_Amin_. Although the value of *Ps* would no longer get more negative due to the slight luminal expansion, the pharyngeal jet with already high kinetic energy (*Pv*) promoted the downstream flow. The narrower the upstream plane_Amin_, the stronger the jet impingement on the slightly expansive pharyngeal wall, the higher force per unit area exerted by the wall on the flow. In addition, the longer the constant ring-shaped constrictive segment, the higher the total frictional loss (such as FTP IV with MO, Figures 4, 5, 7, 8). The constant ring-shaped constrictive segment with the slight expansion might predict transient flow separation and regional small recirculation corresponding to the relatively less inter flow loss. Thus, in level 2, the frictional loss resulted from WSS might still be the major component of pressure loss. In level 3, the rapidly expanded pharyngeal lumen caused a significant flow separation and recirculation region (Figures 7, 9). The bulk of pharyngeal jet flowed within the center of the lumen and toward the posterior pharyngeal wall, contributing to the WSS-associated frictional loss (Figures 7–9). The recirculation at the anterior and lateral part of expanded pharyngeal lumen where flow moved in the upstream direction led to the interior flow loss (Figures 7–9).

For FTP I with MC, the convergent airflow in level 1 (planes 1–44) predicted that the frictional loss related to WSS mainly contributed to the variation of *Pt*’ (−15 Pa, Figure 4). After the plane_Amin_, the rapidly expanded lumen corresponding to the rapid transition from level 2 to level 3 (or directly to level 3) caused flow separation and a larger recirculation region where TKE decreased transiently and increased again (Figures 4, 7). The jet originating from the velopharyngeal constriction almost disappeared at the vicinity of the tip of epiglottis (Figure 5). The variation of *Pt*’ (−40 Pa, Figure 4) in this pharyngeal segment (levels 2 and 3, planes 44–96) could result from both frictional loss and interior flow loss, in which the latter would be found as the primary component (Figures 6, 7). For FTP I with MO, the improved velopharyngeal constriction associated with MO led to a smoother flow through velopharynx into the upper oropharynx and a longer level 1 (planes 1–102) with lower WSS and TKE, which corresponded to the lower *Pt*’ value (<10 Pa, Figures 4–7).

For FTP II with MC, a jet formation (level 1) with a sharp rise in the magnitudes of velocity, WSS, and TKE was attributed to the baseline plane_Amin_ (plane 14) that was close to the top of velopharyngeal lumen (Figures 3–5). In level 2 (planes 14–54), the convergent flow through the long constant constriction led to the significantly higher WSS (Figures 4, 5, 7) with the magnitude more than 1 Pa in each subsegment (subsegs 14–31). Additionally, as shown in Figure 5, a secondary laryngeal jet began to form at the inner surface of the tip of soft palate (plane 44). A repetition of rise in the magnitude of WSS might increase the total frictional loss (Figure 4, planes 38–50). We inferred that the frictional loss resulted from a higher WSS was the major component of the variation of *Pt*’ value (−24 Pa, planes 14–54) in level 2 (Figures 4, 8). In level 3 (planes 54–95), the variation of *Pt*’ value (−14 Pa) in oropharyngeal segment resulted from both frictional loss and interior flow loss. For FTP II with MO, the plane_Amin_ moved downward to the vicinity of the tip of soft palate (Figure 2, plane 54). In level 1, the improved velopharyngeal constriction associated with MO led to a smoother airflow with low WSS and TKE, which corresponded to the low *Pt*’ value (<10 Pa). In level 2, the jet traveled within a longer constriction and a slight expansion of the upper oropharyngeal lumen (planes 54–79) that led to a significantly higher WSS (red and yellow colors in Figure 8). It corresponded to the variation of *Pt*’ value (−14 Pa, Figure 4). In level 3 (planes 79–114), the variation of *Pt*’ value (−14 Pa, Figure 4) in the rapidly expansive pharyngeal segment was attributed to both frictional loss (jet impingement) and interior flow loss (flow separation and recirculation).

For FTP III with MC, the convergent flow in level 1 with the increase of velocity and TKE in the progressive narrowing lumen led to the elevation of WSS (Figure 4, planes 1–47; red color in Figure 8), which corresponded to the frictional loss that might be considered as a major contributor to the variation of *Pt*’ value (−8 Pa). In level 2 (planes 47–68), the slightly expansive lumen led to the small flow separation, while a strong jet impingement on the velopharyngeal wall, which corresponded to the still high WSS (Figures 4, 8). The variation of *Pt*’ value (−14 Pa) in level 2 was also primarily due to the frictional loss associated with WSS. In level 3 (planes 68–91), the magnitude of WSS due to the jet impingement on the posterior pharyngeal wall was stabilized at .5 Pa (Figure 4). Formation of recirculation region due to the expansive oropharyngeal lumen (Figure 7) and the re-increase of TKE (Figure 4) both highlighted that the persistent increase in the variation of *Pt*’ value (−9 Pa) was predominantly comprised of interior flow loss in level 3. For FTP III with MO, the flow field and distribution of CFD endpoints (including the variation of *Pt*’ value) were highly similar to those for FTP III with MC, because there was no obvious change in geometric characteristics from MC to MO (Figures 2–4).

For FTP IV, MO led to a rapidly constrictive lumen with the narrowest plane_Amin_ and caused the strongest jet flow (Figures 2, 3, 5, 7). In level 1 (plane 1–41), the frictional loss related to WSS contributed mainly to the variation of *Pt*’ (−9 Pa). In level 2 (planes 41–53), the strongest jet impingement on the wall of the constant constrictive segment was associated with the still high WSS. The variation of *Pt*’ value (−14 Pa) in the level 2 was also primarily attributed to the frictional loss (Figure 4). In level 3 (planes 53–103), the magnitude of WSS higher than .5 Pa was preserved for a long distance due to the longest jet impingement on the wall of the narrower oropharyngeal lumen (Figures 4, 5, 7, 8). The interior flow loss in level 3 resulted from recirculation formation and flow separation was not a dominant component due to the narrower oropharyngeal lumen (Figures 6, 7). The variation of *Pt*’ value (−20 Pa) in level 3 might be also mainly attributed to the WSS-associated frictional loss (Figure 4). For FTP IV with MC, the relatively minor variation in the luminal diameter of the baseline ROIs predicted a small fluctuation in the resistance to flow, which corresponded to a slight decrease in *Ps* value along the ROIs to drive the flow (Figures 2–4). The larger plane_Amin_ also predicted a remarkably weaker jet formation, less jet impingement, and downstream jet flow separation, which corresponded to the lower variation of *Pt’* value in the overall ROIs (−12 Pa, Figures 4–7).

### Limitations

There were several limitations in the present study. First, the sample size was very small, and only four subjects with different FTP grades were included. Second, the steady-state simulations with constant flow rate boundary conditions were used for CFD analysis. The findings of (Bates et al., 2015) and our preliminary unsteady numerical simulations showed that realistic breathing conditions were cyclic, and the magnitude of CFD endpoints, such as pressure and flow velocity, changed with the flow rate, while the patterns and distribution of flow were similar. Additionally, the flow rate boundary conditions were constant across all subjects irrespective of MC or MO to highlight the effects of the purely anatomical differences on the results. Third, CT scan was performed during wakefulness. The aerodynamic changes corresponding to the geometric variations from MC to MO would be the predictive consequences in subjects with different FTP grades, irrespective of wakefulness or sleep. During CT scan, subjects were instructed to open their mouth in a natural and comfortable state without quantifying the amount of MO. There was no fixed amount of MO that would be appropriate for each subject enrolled (Hu et al., 2018). Fourth, CT images were obtained without considering the respiratory cycle and airway muscle reactions. During CT scan, the flutter of soft palate and blurred border of pharyngeal lumen due to the constant changing respiratory flow might lead to unclear CT images. In such a case, it would always be difficult in the subsequent image processing. Consequently, the results of numerical simulation would be inaccurate and confounding. We always needed to review the images immediately after CT scans. If the pharyngeal wall or soft tissue were fuzzy, CT scan should be performed once again. Although the respiratory cycle was not controlled, we proposed that if a clear serial CT image was obtained, the respiratory cycle might have a much smaller effect on pharyngeal lumen than the action from MC to MO. Last but not the least, it was contrary to the common sense that the pharyngeal airway of subjects with FTP IV had the largest baseline luminal diameters as shown in Figure 2 and Table 2. Previous studies indicated that subjects with a higher FTP grade tended to present with more constrictive pharyngeal airway corresponding to a higher BMI, a larger neck circumference, and a higher severity of OSAHS (Lee et al., 2009; Isono, 2012; Genta et al., 2014; Chen et al., 2019; Xu et al., 2020). For the majority of subjects with FTP IV in our database, the already constrictive baseline pharyngeal lumen would be narrower after MO, especially at the locations of the lower velopharyngeal lumen and the upper oropharyngeal lumen. The narrower pharyngeal lumen reduced the spatial resolution and regional quality of the CT images, caused the ambiguity of soft tissues in determining the boundary between the flow domain and the pharyngeal wall, increased difficulty in the subsequent geometry construction and mesh generation, and influenced the accuracy of numerical simulation. A subject’s 3D upper airway models in the MO position should be first reconstructed to meet the standards of mesh quality, and failure in the CFD analysis may result in exclusion of those models from further study. Due to the inclusion/exclusion criteria and small sample size, the effect of MO on the variation of aerodynamics and pressure loss should be interpreted boldly based on the within-subject comparison (between MC and MO), while prudently when applied to the between-subject comparison (subjects with different FTP grades).

## Conclusion

The variation tendencies of the pharyngeal pressure loss from MC to MO were different in subjects with different FTP grades. MO leading to a narrower plane_Amin_ or/and a longer constant constrictive segment especially in subjects with higher FTP grade might predict that more negative static pressure (*Ps*) would be required to compensate for the higher pharyngeal total pressure loss. A higher *Ps* value acting in the direction towards the interior of the airway could further narrow the pharyngeal lumen and potentially increase the severity of OSAHS in patients with habitual MO during sleep.

## Data Availability

The raw data supporting the conclusion of this article will be made available by the authors, without undue reservation.
